# Multiple doses of adipose tissue‐derived mesenchymal stromal cells induce immunosuppression in experimental asthma

**DOI:** 10.1002/sctm.19-0120

**Published:** 2019-11-20

**Authors:** Ligia L. Castro, Jamil Z. Kitoko, Debora G. Xisto, Priscilla C. Olsen, Herbert L. M. Guedes, Marcelo M. Morales, Miquéias Lopes‐Pacheco, Fernanda F. Cruz, Patricia R. M. Rocco

**Affiliations:** ^1^ Laboratory of Pulmonary Investigation Carlos Chagas Filho Institute of Biophysics, Federal University of Rio de Janeiro Rio de Janeiro Brazil; ^2^ National Institute of Science and Technology for Regenerative Medicine Rio de Janeiro Brazil; ^3^ Laboratory of Clinical Bacteriology and Immunology School of Pharmacy, Federal University of Rio de Janeiro Rio de Janeiro Brazil; ^4^ Laboratory of Glycobiology Carlos Chagas Filho Institute of Biophysics, Federal University of Rio de Janeiro Rio de Janeiro Brazil; ^5^ Laboratory of Cellular and Molecular Physiology Carlos Chagas Filho Institute of Biophysics, Federal University of Rio de Janeiro Rio de Janeiro Brazil

**Keywords:** adipose stem cells, cellular therapy, immunosuppression, lung, mesenchymal stromal cells

## Abstract

In experimental house dust mite (HDM)‐induced allergic asthma, therapeutic administration of a single dose of adipose tissue‐derived mesenchymal stromal cells (MSCs) ameliorates lung inflammation but is unable to reverse remodeling. We hypothesized that multiple doses of MSCs might exert better therapeutic effects by reducing lung inflammation and remodeling but might also result in immunosuppressive effects in experimental asthma. HDM was administered intranasally in C57BL/6 mice. After the last HDM challenge, mice received two or three doses of MSCs (10^5^ cells per day) or saline intravenously. An additional cohort of mice received dexamethasone as a positive control for immunosuppression. Two and three doses of MSCs reduced lung inflammation, levels of interleukin (IL)‐4, IL‐13, and eotaxin; total leukocyte, CD4^+^ T‐cell, and eosinophil counts in bronchoalveolar lavage fluid; and total leukocyte counts in bone marrow, spleen, and mediastinal lymph nodes. Two and three doses of MSCs also reduced collagen fiber content and transforming growth factor‐β levels in lung tissue; however, the three‐dose regimen was more effective, and reduced these parameters to control levels, while also decreasing α‐actin content in lung tissue. Two and three doses of MSCs improved lung mechanics. Dexamethasone, two and three doses of MSCs similarly increased galectin levels, but only the three‐dose regimen increased CD39 levels in the thymus. Dexamethasone and the three‐dose, but not the two‐dose regimen, also increased levels of programmed death receptor‐1 and IL‐10, while reducing CD4^+^CD8^low^ cell percentage in the thymus. In conclusion, multiple doses of MSCs reduced lung inflammation and remodeling while causing immunosuppression in HDM‐induced allergic asthma.

## Graphical abstract

Multiple doses of mesenchymal stromal cells (MSCs) reduced lung inflammation and remodeling. However, three‐dose regimen was more effective at reducing remodeling parameters. Dexamethasone, two and three doses of MSCs similarly increased galectin levels, but only the three‐dose regimen increased CD39 levels in thymus. Dexamethasone and the three‐dose regimen, but not the two‐dose regimen, also increased levels of PD‐1 and IL‐10 in thymus.

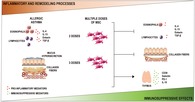

**Significance statement**

Although a single dose of mesenchymal stromal cells (MSCs) administered therapeutically was unable to ameliorate lung remodeling in house dust mite (HDM)‐induced allergic asthma, multiple doses of MSCs effectively reduced both lung inflammation and remodeling, while improving lung mechanics. Nevertheless, multiple doses of MSCs also resulted in immunosuppressive effects. This should be taken into account for future clinical trials in patients with severe asthma.

## 1 INTRODUCTION

Asthma currently affects 1%‐18% of the population in different regions worldwide and represents a growing public health issue. It is a chronic inflammatory disease characterized by immune responses against allergens that result in lung remodeling, airway hyperresponsiveness, and airflow obstruction. Although many patients with asthma respond positively to corticosteroid therapy, with efficient relief of symptoms, it does not reverse established remodeling, and a cohort of patients still experiences poor control of symptoms and exacerbations.^1^, ^2^

Mesenchymal stromal cell (MSC) therapy has demonstrated promising results in several lung disease models, but its impact on airway remodeling has been sometimes controversial.^3^, ^4^, ^5^, ^6^ In models of ovalbumin‐ and *Aspergillus* hyphal extract‐induced allergic asthma, a single dose of MSCs administered either locally or systemically resulted in reduction of both lung inflammation and remodeling.^3^, ^7^, ^8^, ^9^, ^10^ Conversely, in house dust mite extract (HDM)‐induced allergic asthma, a single dose of MSCs reduced some inflammatory parameters but was unable to ameliorate lung function or remodeling.^4^, ^5^ Some reports have indicated that repeated cell‐based therapy yielded better therapeutic effects, either by preventing disease progression or by further reducing inflammation and remodeling, in experimental silicosis and elastase‐induced emphysema.^6^, ^11^ However, the impact of multiple‐dose cell therapy regimens in experimental allergic asthma remains unknown.

We hypothesized that, in experimental allergic asthma, multiple doses of MSCs might exert better therapeutic effects by reducing lung inflammation and remodeling but might also result in immunosuppressive effects. To address the therapeutic potential of a multiple‐dose MSC regimen, this study aimed to comparatively investigate the effects of two and three doses of human adipose tissue‐derived MSCs administered systemically in a model of HDM‐induced allergic asthma. Endpoints of interest included lung mechanics, histology, inflammation, and remodeling; protein levels of selected mediators; leukocyte counts in primary and secondary lymphoid organs; as well as expression of immunosuppression‐associated markers in the thymus.

## 2 MATERIALS AND METHODS

This study was approved by the Ethics Committee of the Federal University of Rio de Janeiro Health Sciences Center (CEUA‐UFRJ: 047/17, Rio de Janeiro, Brazil). All animals received humane care in compliance with the “Principles of Laboratory Animal Care” formulated by the National Society for Medical Research and the *Guide for the Care and Use of Laboratory Animals* prepared by the U.S. National Academy of Sciences.

### 2.1 Isolation and culture of MSCs

MSCs were isolated and cultured as described elsewhere.^3^ Human adipose tissue was obtained from three healthy women (age 21‐45 years) undergoing abdominal plastic surgery at Clementino Fraga Filho Hospital, Federal University of Rio de Janeiro, after Institutional Ethics Committee approval (CEP‐UFRJ: 088/04, Rio de Janeiro, Brazil). Briefly, adipose tissue was digested under agitation with 0.1% type I collagenase (#SRC103, Sigma‐Aldrich, St. Louis, Missouri) at 37°C for 30 minutes. The *pellet* obtained was filtered through 10‐mm nylon membranes and centrifuged twice at 230*g* for 5 minutes. The *pellet* was then resuspended in Dulbecco's modified Eagle's medium (DMEM) (Life Technologies, Grand Island, New York) and cells were counted in a hemocytometer. Cells were cultured in DMEM low‐glucose medium containing 20% fetal bovine serum (FBS, Life Technologies), 100 units/mL penicillin, and 100 μg/mL streptomycin antibiotic solution (Gibco, Albuquerque, New Mexico) at 37°C, in a 5% CO_2_ atmosphere. Nonadherent cells were removed 24 hours after the initial culture. After reaching 80% confluence, cells were detached with TrypLE (Life Technologies) and transferred to new flasks. At the third passage, cell viability, density, and final concentration (1 × 10^5^ viable cells per 50 μL of sterile saline) were determined by trypan blue exclusion in a hemocytometer. The resulting cells were used in experiments.

### 2.2 HDM‐induced allergic asthma and therapeutic protocol

Allergic asthma was induced by HDM exposure as described elsewhere.^4^, ^12^ Briefly, female C57BL/6 mice (age 8‐10 weeks, weight 20‐25 g) were randomly divided into two groups. The HDM group was challenged by intranasal instillation with 25 μg of HDM extract (*Dermatophagoides pteronyssinus*; Greer Laboratories, Lenoir, North Carolina) diluted into 25 μL of PBS, three times a week, for three consecutive weeks, while control (CTRL) animals received 25 μL of sterile PBS under same conditions. One day after the last HDM challenge, the HDM group was further divided to randomly receive either two doses of MSCs (MSC‐2D) (10^5^ AD‐MSC diluted in 50 μL of 0.9% NaCl per dose), three doses of MSCs (MSC‐3D), or three doses of saline (SAL) intravenously, via jugular injection under anesthesia (5% isoflurane). Each dose of MSCs or saline was administered once per day on three consecutive days. Seven days after the last HDM challenge, all animals were euthanized for data analysis (Supporting Information Figure S1). All animals were weighed before and after the end of the protocol.

### 2.3 Lung mechanics

Seven days after the last HDM challenge, the animals were sedated (diazepam 1 mg/kg intraperitoneally), anesthetized (thiopental sodium 20 mg/kg intraperitoneally), tracheotomized, paralyzed (vecuronium bromide, 0.005 mg/kg intravenously), and ventilated with a constant‐flow ventilator (Samay VR15; Universidad de la Republica, Montevideo, Uruguay) set to RR = 100 bpm, tidal volume (V_T_) = 0.2 mL, and fraction of inspired oxygen (FIO_2_) = 0.21. The anterior chest wall was surgically removed and a positive end‐expiratory pressure of 2 cmH_2_O applied. Airflow and tracheal pressure (Ptr) were measured,^3^ and lung mechanics were analyzed by the end‐inflation occlusion method.^13^ In an open chest preparation, Ptr reflects transpulmonary pressure (P_L_). Briefly, after end‐inspiratory occlusion, there is an initial rapid decline in P_L_ (ΔP1,L) from the preocclusion value down to an inflection point (Pi), followed by a slow pressure decay until a plateau is reached. This plateau corresponds to the elastic recoil pressure of the lung (Pel). ΔP1,L selectively reflects the pressure used to overcome the airway resistance. Static lung elastance (Est,L) was determined by dividing Pel by V_T_. Lung mechanics measurements were obtained 10 times in each animal.^3^ All data were analyzed using ANADAT software (RHT‐InfoData, Inc, Montreal, Quebec, Canada).

### 2.4 Collection and processing of BALF and tissues

Bronchoalveolar lavage fluid (BALF) was collected with a polyethylene cannula which was inserted into the trachea and through which a total volume of 1 mL of PBS containing 10 mM EDTA was instilled and subsequently aspirated. BALF was centrifuged at 300*g* for 10 minutes at 4°C. The supernatant was removed, and the *pellet* resuspended in 250 μL of PBS.

The left lung was removed, frozen immediately in liquid nitrogen, and stored at −80°C for molecular analysis, while the right lung was stored in 4% paraformaldehyde solution for histological analyses.

Mediastinal lymph nodes, femoral bone marrow, spleen, and thymus were also removed and macerated with 1 mL PBS. Mediastinal lymph nodes and thymus were weighed before maceration. All samples were stored at 4°C.

### 2.5 Total and differential cell counts

For total cell counts, all samples were quantitated in a hemocytometer under light microscopy after dilution of the samples in Türk solution. Cell suspensions from BALF and thymus were blocked with anti‐CD16/32 (eBioscience, San Diego, California) and then stained with specific primary antibodies. T cells were characterized by using monoclonal anti‐mouse CD3 (Pe‐Cy5‐labeled), CD4 (FITC‐labeled), and CD8 (FITC‐labeled) antibodies, while eosinophils were characterized by staining with the anti‐mouse Siglec‐F antibody (PE‐labeled; BD Pharmingen, San Diego, California) in the polymorphonuclear‐gated populations. All data were acquired in a FACSCalibur flow cytometer (BD Biosciences Immunocytometry Systems, San Jose, California) and analyzed using FlowJo X 10.0.7 software (Tree Star Inc, Ashland, Oregon).

### 2.6 Lung histology

Sections (4 μm thick) were cut, deparaffinized, and stained for histological analysis. For quantification of collagen fibers in alveolar septa, sections were stained with Sirius Red dissolved in saturated picric acid for subsequent analysis through polarized‐light optical microscopy (BX51, Olympus Latin America Inc, Miami, Florida), under ×400 magnification. The area occupied by fibers was determined by digital densitometric recognition (Image‐Pro Plus 7.1 Software, Media Cybernetics, Silver Spring, Maryland) and divided by the area of alveolar septa. Results were expressed as the fractional area occupied by collagen fibers.^3^ For quantification of α‐smooth muscle actin (α‐actin), immunohistochemistry was performed using a monoclonal antibody (α‐actin; Dako, Carpinteria, California) at a 1:500 dilution. Analysis was performed by applying the point‐counting technique.^14^ Using a 121‐point grid, the volume proportion of α‐actin was calculated as a ratio of points falling on actin‐stained *vs* non‐stained tissue. To determine inflammation in the lung parenchyma, the slices were stained with hematoxylin and eosin. Inflammatory changes were graded according to a semiquantitative scoring system as absent (score 0), mild (score 1‐2), moderate (score 3), or severe (score 4) by two blinded researchers. The following scoring criteria were adopted: 0, no evidence of inflammation; 1, few inflammatory cells in the peribronchial area; 2, a ring of inflammatory cells (one cell layer deep); 3, a ring of inflammatory cells two to four cells deep; and 4, a ring of inflammatory cells more than four cells deep.^15^

### 2.7 Enzyme‐linked immunosorbent assay (ELISA)

Lung tissue and thymus homogenates were used for mediator quantifications. Briefly, the right lung was isolated, homogenized in lysis buffer solution, centrifuged (600*g* for 5 minutes and 10 000*g* for 10 minutes), and the resulting supernatant assayed. Total protein content was measured by Bradford's reagent (Sigma‐Aldrich). Protein levels of IL‐4, IL‐13, eotaxin, IL‐10, transforming growth factor (TGF)‐β (lung tissue), and programmed cell death protein‐1 (PD‐1) and IL‐10 (thymus) were evaluated by ELISA (BioLegend, San Diego, California) using matched antibodies in accordance with manufacturer instructions. The final concentration was normalized to total protein content and expressed as pg/mg.

### 2.8 Analyses of immunosuppression‐associated markers

Thymic tissue was lysed for RNA extraction by the RNeasy Plus Mini Kit (Qiagen, Valencia, California) in accordance with manufacturer instructions. The total RNA concentration was measured by spectrophotometry in a Nanodrop ND1000 system, and the first‐strand cDNA was synthesized from total RNA using the High‐Capacity cDNA Reverse Transcription Kit (Applied Biosystems, Foster City, California). Relative mRNA levels were measured with Bryt Green (Promega, Fitchburg, Wisconsin) using a Mastercycler ep realplex PCR system (Eppendorf, Hamburg, Germany). All experiments were performed in triplicate. The relative level of each gene was normalized to the housekeeping gene acidic ribosomal phosphoprotein P0 (*36B4*) and expressed as the fold change relative to the CTRL group using the 2^−ΔΔ^ *Ct* method, where Δ*Ct* = *Ct*_(target gene)_ − *Ct*_(housekeeping gene)._ 16 The mRNA expression of the following genes was analyzed: indoleamine 2,3‐dioxygenase (IDO)‐2, CD39, galectin, cytotoxic T‐lymphocyte‐associated antigen (CTLA)‐4, PD‐1, and IL‐10. The sequences of PCR primers can be found in Supporting Information Table S1.

### 2.9 Statistical analysis

Differences among groups were assessed using one‐way ANOVA followed by Bonferroni's test or an unpaired Student's *t* test. Data were expressed as mean ± SD. All tests were performed using the Prism 6.07 software package (GraphPad Software Inc, La Jolla, California), and statistical significance was established at *P* < .05.

## 3 RESULTS

### 3.1 Multiple doses of MSCs reduced lung inflammation in HDM‐induced allergic asthma

**Figure 1:**
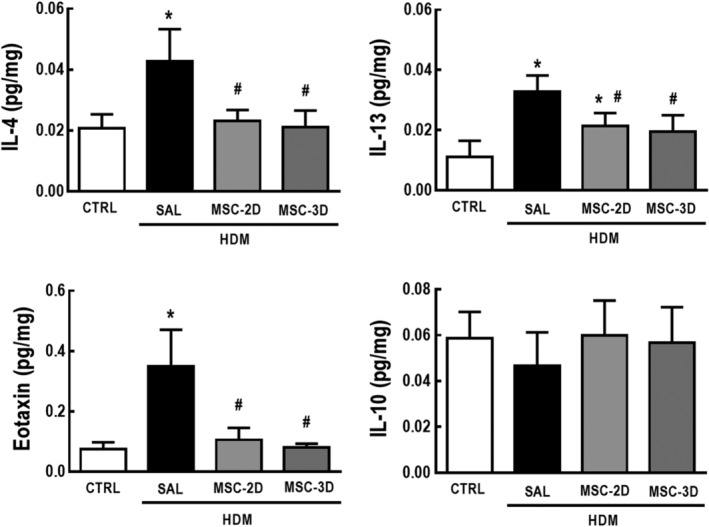
Multiple doses of mesenchymal stromal cells (MSCs) reduced protein levels of lung inflammation‐associated mediators in house dust mite (HDM)‐induced allergic asthma. Protein levels of interleukin (IL)‐4, IL‐13, eotaxin, and IL‐10 in lung tissue homogenates. CTRL, mice challenged with saline and treated with saline. HDM‐SAL, mice challenged with HDM and treated with saline. HDM‐MSC‐2D, mice challenged with HDM and treated with two doses of MSCs (10^5^ cells per dose). HDM‐MSC‐3D, mice challenged with HDM and treated with three doses of MSCs. Data are presented as means ± SD of sox animals per group. *Significantly different from CTRL (*P* < .05). ^#^Significantly different from HDM‐SAL (*P* < .05)

Protein levels of IL‐4, IL‐13, and eotaxin were higher in lung tissue homogenates of HDM‐SAL compared to CTRL mice. Both two and three doses of MSCs significantly reduced protein levels of these inflammatory mediators. No differences were observed in protein levels of IL‐10 among the experimental groups (Figure 1).

**Figure 2:**
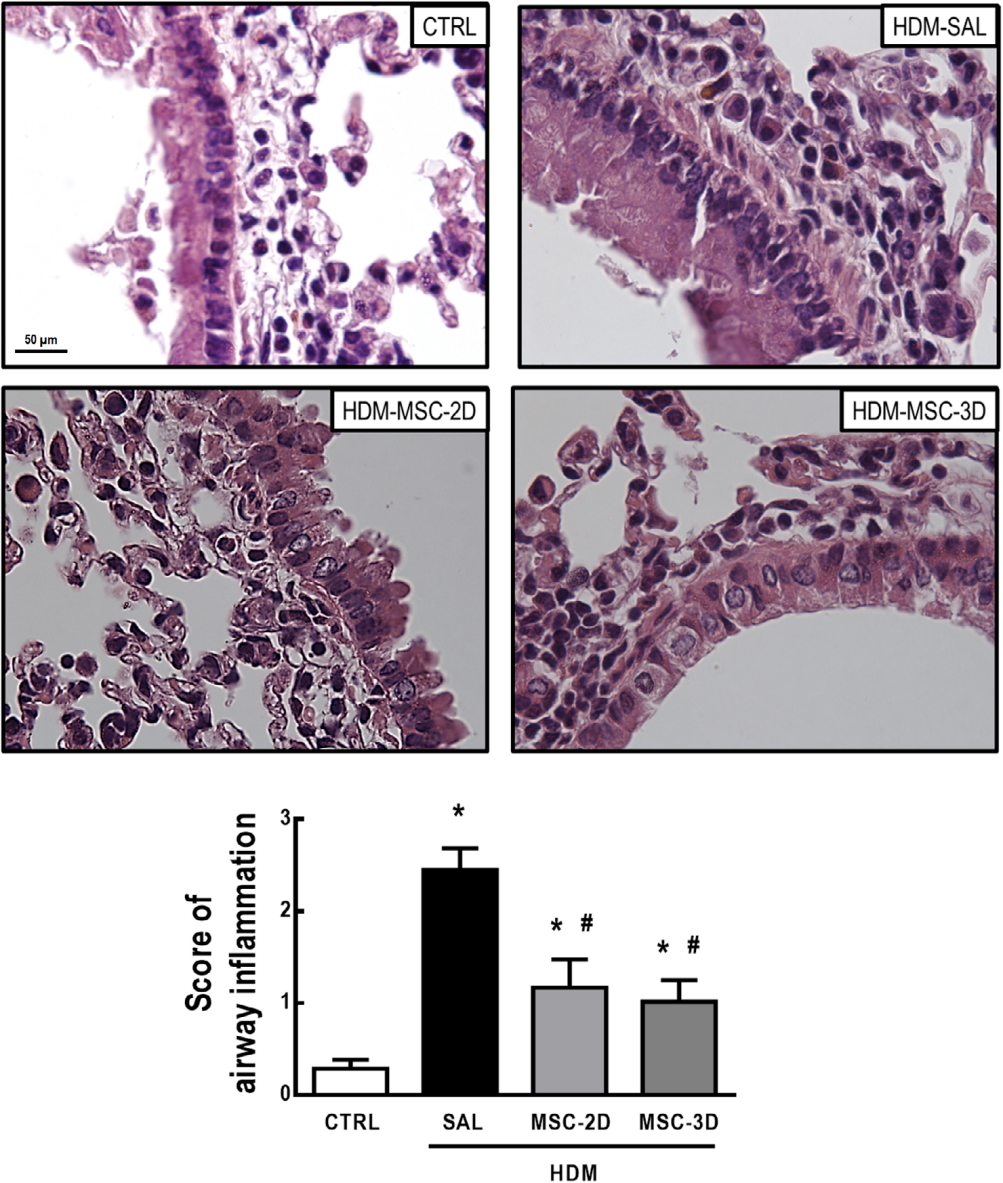
Multiple doses of mesenchymal stromal cells (MSCs) reduced lung inflammation in house dust mite (HDM)‐induced allergic asthma. Representative photomicrographs of lung tissue and cell infiltration score in peribronchial areas. CTRL, mice challenged with saline and treated with saline. HDM‐SAL, mice challenged with HDM and treated with saline. HDM‐MSC‐2D, mice challenged with HDM and treated with two doses of MSCs (10^5^ cells per dose). HDM‐MSC‐3D, mice challenged with HDM and treated with three doses of MSCs. Data are presented as means ± SD of six animals per group. *Significantly different from CTRL (*P* < .05). ^#^Significantly different from HDM‐SAL (*P* < .05)

The HDM‐SAL group demonstrated increased cell counts in peribronchial areas, with moderate lung inflammation scores compared to CTRL mice. Both two and three doses of MSCs reduced cell infiltration in peribronchial areas to counts consistent with a mild lung inflammation score (Figure 2).

**Figure 3:**
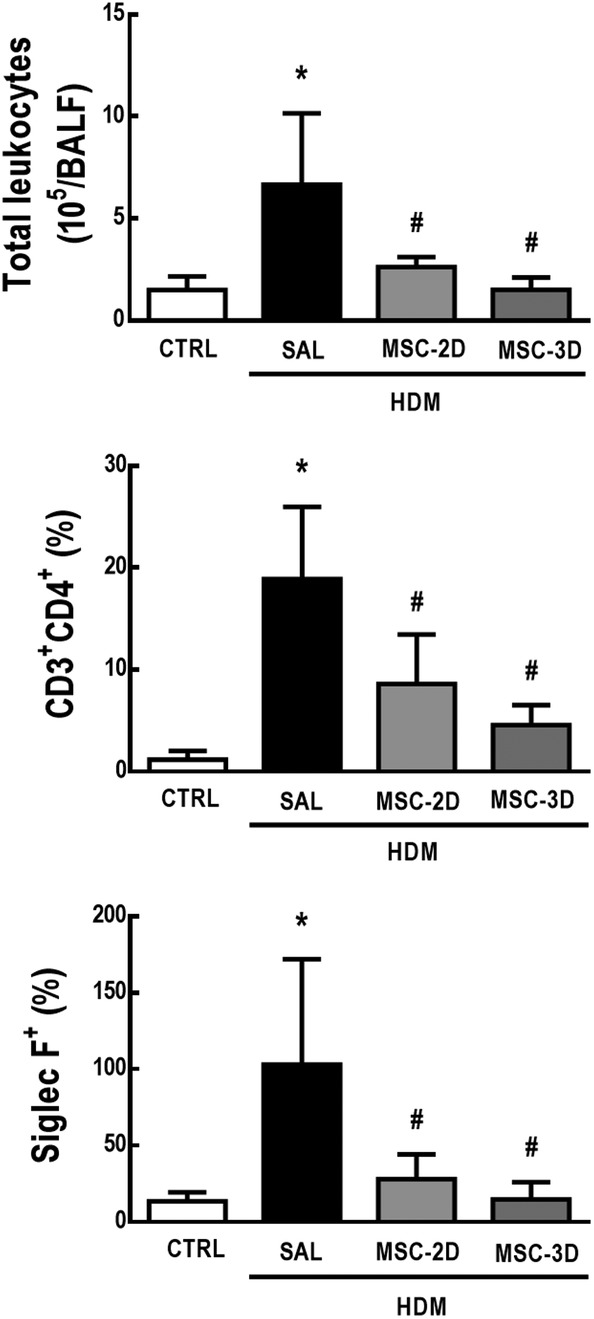
Multiple doses of mesenchymal stromal cells (MSCs) reduced total and differential cell counts in bronchoalveolar lavage fluid (BALF) in house dust mite (HDM)‐induced allergic asthma. Number of total leukocytes, CD4^+^ T‐cells, and eosinophils in the BALF. CTRL, mice challenged with saline and treated with saline. HDM‐SAL, mice challenged with HDM and treated with saline. HDM‐MSC‐2D, mice challenged with HDM and treated with two doses of MSCs (10^5^ cells per dose). HDM‐MSC‐3D, mice challenged with HDM and treated with three doses of MSCs. Data are presented as means ± SD of six animals per group. *Significantly different from CTRL (*P* < .05). ^#^Significantly different from HDM‐SAL (*P* < .05)

Compared to CTRL mice, the HDM‐SAL group demonstrated an increase in total and differential cell counts in the BALF. Both two and three doses of MSCs were able to reduce total leukocyte, CD4^+^ T‐cell, and eosinophil counts (Figure 3; Supporting Information Figure S2).

### 3.2 Three doses of MSCs led to further reduction of lung remodeling compared to the two‐dose regimen

**Figure 4:**
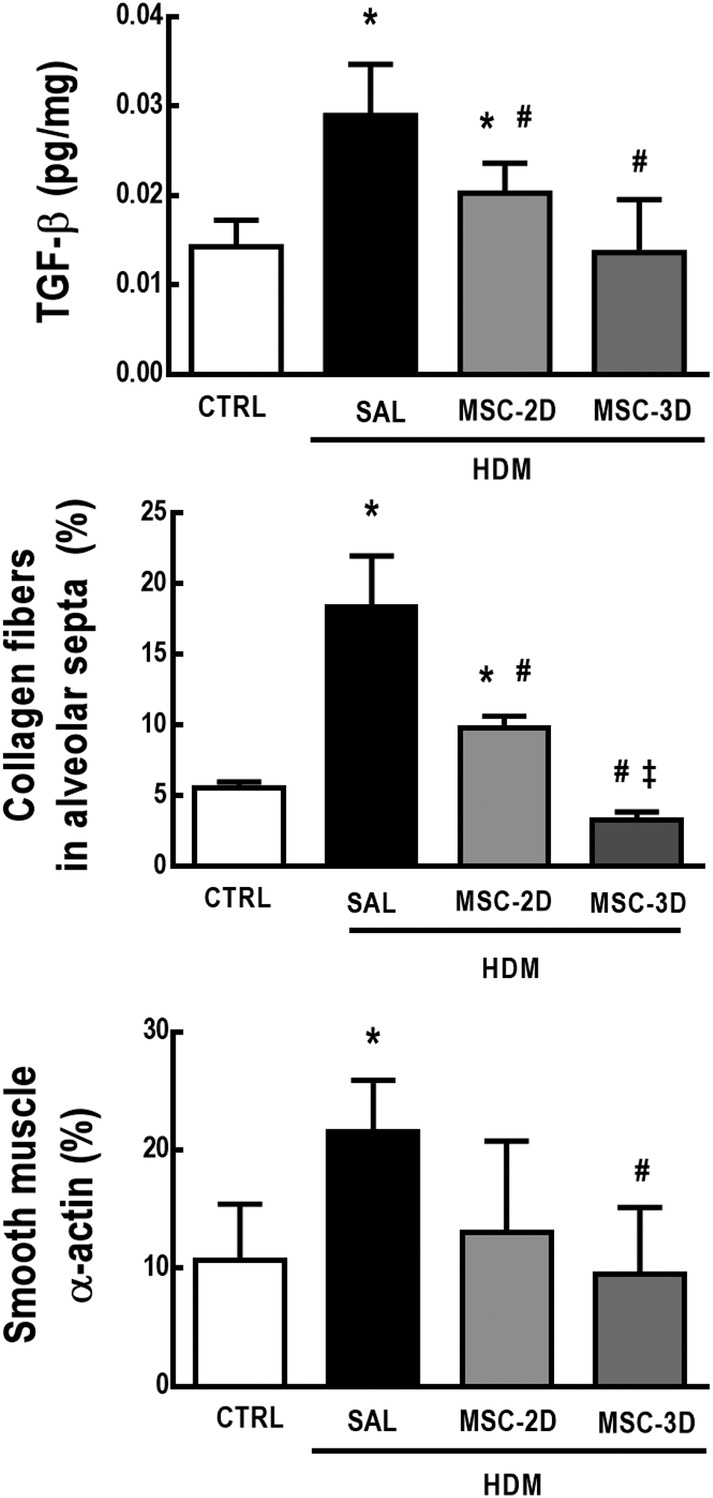
A three‐dose mesenchymal stromal cell (MSC) regimen led to further reduction of lung remodeling in house dust mite (HDM)‐induced allergic asthma than a two‐dose regimen. Protein levels of transforming growth factor (TGF)‐β; collagen fiber content in alveolar septa and percentage of α‐actin content in lung tissue. CTRL, mice challenged with saline and treated with saline. HDM‐SAL, mice challenged with HDM and treated with saline. HDM‐MSC‐2D, mice challenged with HDM and treated with two doses of mesenchymal stromal cells (MSCs) (10^5^ cells per dose). HDM‐MSC‐3D, mice challenged with HDM and treated with three doses of MSCs. Data are presented as means ± SD of six animals per group.*Significantly different from CTRL (*P* < .05). ^#^Significantly different from HDM‐SAL (*P* < .05). ^‡^Significantly different from HDM‐MSC‐2D (*P* < .05)

HDM‐SAL animals demonstrated an increase in TGF‐β levels, as well as in the amount of collagen fibers and α‐actin in alveolar septa compared to CTRL mice. Both two and three doses of MSCs reduced TGF‐β levels and collagen fiber content in lung tissue; however, the three‐dose regimen was more effective, reducing these parameters to CTRL‐comparable levels. The three‐dose regimen, but not two doses of MSCs, reduced α‐actin content in alveolar septa (Figure 4).

### 3.3 Multiple doses of MSCs led to improvements in lung mechanics parameters

**Figure 5:**
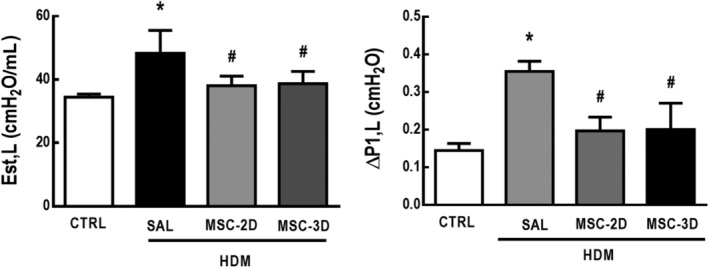
Multiple doses of mesenchymal stromal cells (MSCs) led to improvements in lung mechanics. Static lung elastance (Est,L) and resistive pressures (ΔP1,L). CTRL, mice challenged with saline and treated with saline. House dust mite (HDM)‐SAL, mice challenged with HDM and treated with saline. HDM‐MSC‐2D, mice challenged with HDM and treated with two doses of MSCs (10^5^ cells per dose). HDM‐MSC‐3D, mice challenged with HDM and treated with three doses of MSCs. Data are presented as means ± SD of six animals per group. *Significantly different from CTRL (*P* < .05). ^#^Significantly different from HDM‐SAL (*P* < .05)

HDM‐SAL mice demonstrated higher Est,L and ΔP1,L compared to the CTRL group (1.4‐ and 2.5‐fold increase, respectively). Both two and three doses of MSCs reduced these parameters to CTRL‐comparable levels (Figure 5).

### 3.4 Multiple doses of MSCs reduced total leukocyte counts in bone marrow, spleen, and mediastinal lymph nodes, but not in the thymus

Saline *vs* HDM challenge and treatment with saline *vs* multiple doses of MSCs had no effect on body weight (Supporting Information Table S2). Nevertheless, relative thymus weight was increased in HDM‐SAL compared to CTRL mice. Both the two‐dose and three‐dose MSC regimens reduced relative thymus weight to CTRL‐comparable levels. No differences were observed in relative mediastinal lymph node weight among the experimental groups (Supporting Information Figure S3).

**Figure 6:**
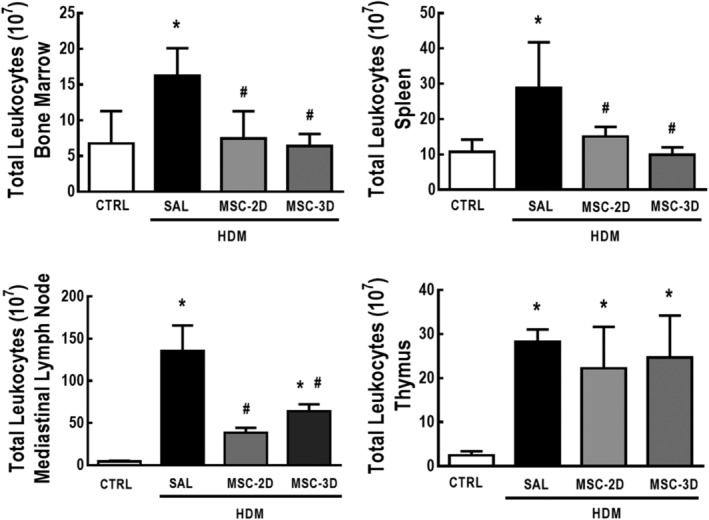
Multiple doses of mesenchymal stromal cells (MSCs) reduced total leukocyte counts in lymphoid organs in house dust mite (HDM)‐induced allergic asthma. Total leukocyte counts in bone marrow, spleen, mediastinal lymph nodes, and thymus. CTRL, mice challenged with saline and treated with saline. HDM‐SAL, mice challenged with HDM and treated with saline. HDM‐MSC‐2D, mice challenged with HDM and treated with two doses of MSCs (10^5^ cells per dose). HDM‐MSC‐3D, mice challenged with HDM and treated with three doses of MSCs. Data are presented as means ± SD of six animals per group. *Significantly different from CTRL (*P* < .05). ^#^Significantly different from HDM‐SAL (*P* < .05)

HDM‐SAL animals demonstrated increased total leukocyte counts in bone marrow, spleen, mediastinal lymph nodes, and thymus compared to CTRL mice. Both the two‐dose and three‐dose MSC regimens reduced total leukocyte counts in bone marrow, spleen, and mediastinal lymph nodes, but not in the thymus (Figure 6). To rule out any hypothesis that multiple manipulations for the intravenous administration of MSCs could cause stress and itself induces an effect on lymphoid organs, a separate cohort of mice (SHAM) was administered a single sham challenge with saline, while the CTRL group was challenged with saline and treated with saline for three consecutive days in parallel to the remaining experimental groups. CTRL and SHAM animals demonstrated no significant differences in total cell counts in bone marrow (*P* = .4420), spleen (*P* = .1318), or thymus (*P* = .7733), nor any other physiological alteration.

### 3.5 Multiple doses of MSCs differentially modulated percentage of T‐cell subpopulations while increasing expression of immunosuppressive markers in the thymus

To analyze whether multiple doses of MSCs may result in increased expression of immunosuppressive markers, a cohort of mice was treated with dexamethasone (DEXA; 1 mg/kg, 3 consecutive days) after the last challenge with HDM, as a positive control for immunosuppression.

Compared to both CTRL and HDM‐SAL groups, HDM‐DEXA mice demonstrated an increased percentage of CD4^+^CD8^+^ cells, as well as a significant reduction in the percentage of CD4^+^CD8^low^ and CD4^+^CD8^−^ cells. The HDM‐MSC‐3D group also demonstrated an increased percentage of CD4^+^CD8^+^ cells and a reduction in the percentage of CD4^+^CD8^low^ cells in comparison to HDM‐SAL mice. The HDM‐MSC‐2D group demonstrated a trend toward reduction of CD4^+^CD8^low^ cell counts, but the difference was not statistically significant in comparison to the CTRL group (*P* = .0656) (Supporting Information Table S3).

**Figure 7:**
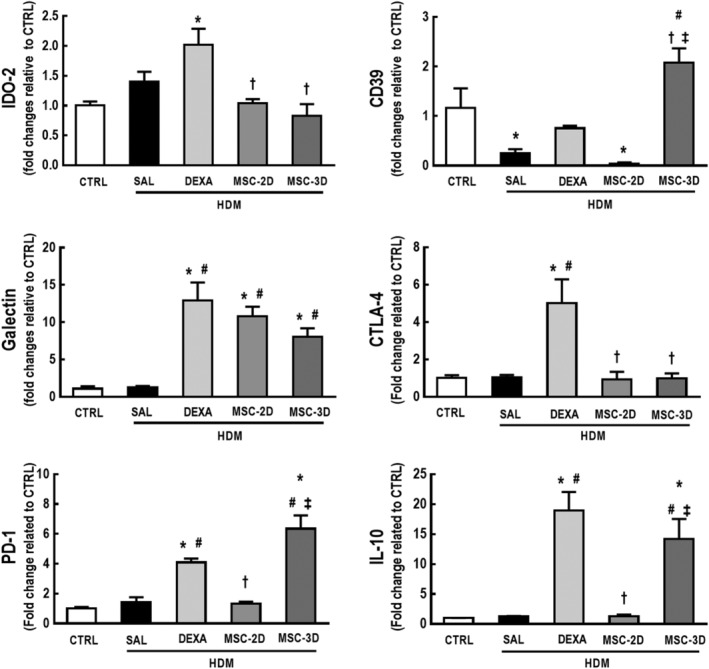
Dexamethasone therapy and multiple doses of mesenchymal stromal cells (MSCs) differentially modulated expression levels of immunosuppression‐associated markers in the thymus of house dust mite (HDM)‐induced allergic asthma. Expression levels of indoleamine 2,3‐dioxygenase (IDO)‐2, CD39, galectin, cytotoxic T‐lymphocyte‐associated antigen (CTLA)‐4, programmed death receptor (PD)‐1, and interleukin (IL)‐10 in the thymic tissue. CTRL, mice challenged with saline and treated with saline. HDM‐SAL, mice challenged with HDM and treated with saline. HDM‐DEXA, mice challenged with HDM and treated with dexamethasone. HDM‐MSC‐2D, mice challenged with HDM and treated with two doses of MSCs (10^5^ cells per dose). HDM‐MSC‐3D, mice challenged with HDM and treated with three doses of MSCs. Data are presented as means ± SD of six animals per group. *Significantly different from CTRL (*P* < .05). ^#^Significantly different from HDM‐SAL (*P* < .05). ^†^Significantly different from HDM‐DEXA (*P* < .05). ^‡^Significantly different from HDM‐MSC‐2D (*P* < .05)

The CTRL and HDM‐SAL groups had similar levels of IDO‐2, galectin, CTLA‐4, PD‐1, and IL‐10, while levels of CD39 were reduced in HDM‐SAL compared to CTRL animals. Levels of galectin, CTLA‐4, PD‐1, and IL‐10 were increased in HDM‐DEXA in comparison to both CTRL and HDM‐SAL. HDM‐DEXA animals also demonstrated increased levels of IDO‐2 compared to CTRL. Two doses of MSCs only increased levels of galectin, whereas the three‐dose MSC regimen increased levels of galectin, PD‐1, and IL‐10 compared to both CTRL and HDM‐SAL. CD39 levels were also increased in HDM‐MSC‐3D compared to HDM‐SAL, HDM‐DEXA, and HDM‐MSC‐2D (Figure 7). Protein levels of PD‐1 and IL‐10 were also increased in thymus homogenates from HDM‐DEXA and HMD‐MSC‐3D animals compared to CTRL, HMD‐SAL, and HMD‐MSC‐2D (Supporting Information Figure S4).

## 4 DISCUSSION

In this study, multiple doses of MSCs resulted in a significant reduction of both lung inflammation and remodeling, and improvement of lung function in a model of HDM‐induced allergic asthma. Additionally, multiple doses of MSCs reduced total leukocyte counts in bone marrow, spleen, and mediastinal lymph nodes, while modulating T‐cell subpopulations and enhancing expression levels of immunosuppression‐associated mediators in the thymus.

HDM is present in almost all environments and is the most common allergen implicated in the onset of human asthma, affecting approximately 85% of asthmatic patients globally.^17^, ^18^ In experimental models, HDM closely reproduces several hallmark features of human disease, including the inflammatory process with Th2 and eosinophilic responses and remodeling of both airways and lung parenchyma.^4^, ^12^, ^19^ In this study, MSCs were administered intravenously 24 hours after the last HDM challenge in order to reproduce a clinically relevant situation, as lung inflammation and remodeling were established and lung function was impaired. Although bone marrow‐derived MSCs have demonstrated therapeutic effects in this model of HDM‐induced allergic asthma,^4^, ^19^ the availability of these cells is limited in the clinical scenario, since an invasive harvesting procedure is required. Therefore, herein we used cells collected from human adipose tissue, which is an interesting source of MSCs, as they can be easily obtained by liposuction. Furthermore, adipose tissue is estimated to contain a greater number of MSCs compared to bone marrow, and these cells appear to be expandable to a higher number of passages,^20^, ^21^ thus providing attractive advantages for use in a multiple‐dose regimen.

In previous studies, a single dose of MSCs administered prophylactically or therapeutically reduced lung influx of inflammatory cells, airway hyperresponsiveness, and mucus hypersecretion in models of ovalbumin‐induced allergic asthma.^3^, ^7^, ^8^, ^22^, ^23^ In HDM‐induced allergic asthma, a single dose of MSCs administered prophylactically also prevented inflammation by modulating epithelial cell activation.^24^ However, effects on the inflammatory process were only marginal, with no improvements in lung function and remodeling when MSCs were administered therapeutically.^4^, ^5^ In this study, both two and three doses of MSCs reduced inflammatory cell counts in peribronchial areas as well as total leukocyte, CD4^+^ T‐cell, and eosinophil counts in the BALF. Multiple doses of MSCs also resulted in improvement of lung mechanics. Either two or three doses of MSCs reduced protein levels of IL‐4, IL‐13, and eotaxin in lung tissue, although protein levels of IL‐10 remained unaltered. Notably, a single dose of bone marrow‐derived MSCs, but not adipose tissue‐derived MSCs, increased IL‐10 levels in lung tissue in experimental allergic asthma,^4^, ^5^ which suggests that the multiple‐dose regimen of adipose tissue‐derived MSCs suppressed Th2 and eosinophilic responses by an IL‐10‐independent mechanism.

IL‐4 and IL‐13 contribute not only to the inflammatory process but also to lung remodeling, as they can induce epithelial cell apoptosis, fibroblast proliferation, airway hyperresponsiveness, and mucus hypersecretion.^25^, ^26^ TGF‐β also participates in the remodeling process by increasing extracellular matrix deposition and promoting differentiation of fibroblasts to myofibroblasts, as reflected by increased smooth muscle α‐actin content.^26^, ^27^ Although a single dose of MSCs was unable to reverse lung remodeling in this model of HDM‐induced allergic asthma,^4^ our present findings demonstrate that multiple doses of MSCs can exert this effect. In fact, three doses of MSCs were even more effective than a two‐dose regimen, thus reducing collagen fiber and smooth muscle α‐actin content in alveolar septa, and protein levels of IL‐13 and TGF‐β to CTRL‐comparable levels. Other studies have also demonstrated better effects on remodeling after repeated cell‐based therapy in distinct models of lung injury,^6^, ^11^ indicating that more than one dose of MSCs possibly induces a more organized and effective re‐epithelization. Furthermore, multiple doses of MSCs resulted in prolonged therapeutic benefits in a non‐obese mouse model with severe diabetes^28^ and in the SOD1^G93A^ mouse model of amyotrophic lateral sclerosis.^29^

Although the mechanisms by which MSCs induce therapeutic actions are not entirely elucidated, these cells have the ability to promote immunomodulation.^3^, ^9^, ^10^, ^23^, ^30^ In this study, multiple doses of MSCs alleviated lung inflammation, which may also correlate with the reduction in total leukocyte counts in the bone marrow, spleen, and mediastinal lymph nodes. As lymphocytes traffic between lymphoid organs during the differentiation process, MSCs may block their production and/or maturation,^31^, ^32^ thus inhibiting the recruitment of inflammatory cells into the lungs. In a model of elastase‐induce emphysema, repeated administration of MSCs also resulted in immunosuppressive effects on lymphoid organs.^6^ Nevertheless, HDM mice demonstrated a reduction in relative thymus weight, but not in total leukocyte counts in this organ, after multiple doses of MSCs. Therefore, we further investigated the impact of multiple doses of MSCs on lymphocyte subpopulations, as the thymus plays a critical role in T‐cell differentiation and maturation. Furthermore, an additional group of HDM mice was treated with dexamethasone, a recommended therapy for asthmatic patients, as a positive control.^2^, ^12^ Although two doses of MSCs elicited no significant change in percentages of T‐cell subpopulations in comparison to the CTRL and HDM‐SAL groups, both the three‐dose MSC regimen and dexamethasone therapy reduced the percentage of CD4^+^CD8^low^ cells, and dexamethasone also reduced the percentage of CD4^+^CD8^−^ cells in the thymus of HDM mice. It is possible that immunosuppressive events may occur by increasing the number of doses of MSCs, since previous studies have demonstrated that human MSCs may migrate to the thymus after administration and prevent lymphocyte differentiation,^31^, ^32^ or may even promote survival of immature T cells in a quiescent state.^33^, ^34^ These phenomena could account for the unchanged number of leukocytes in the thymus after MSC therapy. Dexamethasone therapy also reduced the percentage of CD4^−^CD8^+^ cells in the thymus, as reported elsewhere.^35^ Multiple doses of MSCs resulted in inhibition of CD4^+^ T‐cell maturation, but had less effect on CD8^+^ T‐cell development.

MSC‐induced immunosuppression has been reported in models of autoimmune disorders and in early‐stage clinical trials, with some promising results for the treatment of graft *vs* host disease, multiple sclerosis, systemic lupus erythematosus, and other conditions.^36^ Nevertheless, this is the first study demonstrating that multiple doses of MSCs may induce immunosuppressive effects in experimental allergic asthma. In addition to its impact on T‐cell subpopulations, the three‐dose MSC regimen also increased expression of immunosuppression‐associated markers in the thymus. Both multiple doses of MSCs and dexamethasone increased expression of galectin, which can prevent T‐lymphocyte proliferation by binding to the T‐cell immunoglobulin and mucin domain (TIM)‐3 receptor.^37^, ^38^ It also plays a key role in the generation of tolerogenic DCs^39^ and in regulatory T‐cell (Treg) activity.^40^ Three doses of MSCs and dexamethasone also increased expression of PD‐1 and IL‐10. While MSCs can produce PD‐1 ligands to suppress CD4^+^ T‐cell proliferation by arresting the cell cycle,^41^ IL‐10 can act directly on the CD28 signaling pathway that leads to T‐cell anergy.^42^ Notably, three doses of MSCs and dexamethasone had different impact on the expression of IDO‐2, CD39, and CTLA‐4. Dexamethasone increased expression levels of CTLA‐4, a costimulatory molecule that induces negative effects on T‐cell activation when bound to CD80/CD86,^43^ and IDO‐2, a regulator of IDO‐1 that stimulates T‐cell apoptosis by depleting tryptophan.^44^ On the other hand, three doses of MSCs resulted in increased expression levels of CD39, which induces suppressive effects on activated T‐cells by increasing adenosine production.^45^ Such differences suggest that multiple doses of MSCs and dexamethasone may induce immunosuppressive effects by distinct mechanisms. Even though MSC‐induced immunosuppression may reduce tissue injury in uncontrolled inflammatory disorders, possible complications should be taken into account, as immunosurveillance against opportunistic pathogens would be reduced and patients with severe asthma are already clinically debilitated.

This study has some limitations that should be addressed. Two and three doses of MSCs were selected rather than four or more, since further increasing the number of MSC doses would have subjected animals to additional anesthesia procedures. Such repeated use of anesthetic agents might hinder the possible beneficial effects of MSCs. Additionally, more experimental groups would be required, thus adding more variables to the comparative statistical analysis. Further studies are warranted to comparatively evaluate the short‐ and long‐term effects of multiple doses.

## 5 CONCLUSION

In HDM‐induced allergic asthma, multiple doses of MSCs were associated with reductions in inflammation and remodeling, while resulting in T‐cell immunosuppression. These findings should be borne in mind for future clinical trials.

## AUTHOR CONTRIBUTIONS

L.L.C.: conducted the experiments and study, contributed to data collection and analysis, interpreted the data, wrote the first draft; J.Z.K., D.G.X., P.C.O., H.L.M.G.: conducted the experiments and contributed to data collection and analysis; M.M.M. provided critical revisions for important intellectual content; M.L.P.: interpreted the data, wrote and edited the manuscript; F.F.C. and P.R.M.R. contributed to idea, conception and designed of study, interpreted the data, edited and reviewed the manuscript. All the authors approved the final version of the manuscript.

## CONFLICT OF INTEREST

The authors declared no potential conflict of interest.

## Supporting information


**Supporting Information Figure S1** Experimental design. Female C57BL/6 mice were randomly divided into 2 groups. CTRL group was challenged with 25 μL of saline intranasally (i.n.) 3 times a week for 3 weeks and HDM group received 25 μg of HDM diluted in saline (25 μL). The HDM group was then treated with saline (50 μL) for 3 consecutive days after the last challenge, or 2 or 3 doses of 10^5^ adipose tissue (AD) derived‐MSC diluted in saline (50 μL) for 2 or 3 consecutive days after the last challenge (MSC‐2D and MSC‐3D, respectively). All treatments were administered intravenously. Seven days after the last challenge, the animals were euthanized for data acquisition.

**Supporting Information Figure S2** Representative histogram with the gating strategy used to quantify CD4+ T‐cells (top) and eosinophils (bottom) in bronchoalveolar lavage fluid.

**Supporting Information Figure S3** Relative weight of mediastinal lymph nodes and thymus. Data are presented as means + SD of 6 animals/group. SAL: mice challenged with HDM and treated with saline. MSC‐2D and MSC‐3D: mice challenged with HDM and treated with 2 or 3 doses of AD‐MSCs, respectively. * Significantly different from CTRL (*P* < 0.05). # Significantly different from SAL (*P* < 0.05).

**Supporting Information Figure S4** Dexamethasone therapy and three doses of MSCs modulated protein levels of programmed death receptor (PD)‐1, and interleukin (IL)‐10 in the thymic tissue of animals with HDM‐induced allergic asthma. CTRL, mice challenged with saline and treated with saline. HDM‐SAL, mice challenged with HDM and treated with saline. HDM‐DEXA, mice challenged with HDM and treated with dexamethasone. HDM‐MSC‐2D, mice challenged with HDM and treated with two doses of MSCs (10^5^ cells per dose). HDM‐MSC‐3D, mice challenged with HDM and treated with three doses of MSCs. Data are presented as means ± SD of 6 animals/group. *Significantly different from CTRL (*P* < 0.05). ^#^Significantly different from HDM‐SAL (P < 0.05). ^†^Significantly different from HDM‐DEXA (P < 0.05). ^‡^Significantly different from HDM‐MSC‐2D (P < 0.05).

**Table S1** Sequence of PCR primers

**Table S2** Body weight of animals (g) before and after HDM‐induced allergic asthma and therapeutic protocol

**Table S3** T‐cell subpopulations in the thymus

## Data Availability

The data that support the findings of this study are available from the corresponding author upon reasonable request.
