# Supplementary material for: Multiple doses of adipose tissue‐derived mesenchymal stromal cells induce immunosuppression in experimental asthma
Source: Stem Cells Transl Med. 2019 Nov 20;9(2):250–60. doi: 10.1002/sctm.19-0120 (PMC6988761; doi:10.1002/sctm.19-0120)
Supplement: Supplementary file 1 — Supporting Information Figure S1 Experimental design. Female C57BL/6 mice were randomly divided into 2 groups. CTRL group was challenged with 25 μL of saline intranasally (i.n.) 3 times a week for 3 weeks and HDM group received 25 μg of HDM diluted in saline (25 μL). The HDM group was then treated with saline (50 μL) for 3 consecutive days after the last challenge, or 2 or 3 doses of 105 adipose tissue (AD) derived‐MSC diluted in saline (50 μL) for 2 or 3 consecutive days after the last challenge (MSC‐2D and MSC‐3D, respectively). All treatments were administered intravenously. Seven days after the last challenge, the animals were euthanized for data acquisition. [file SCT3-9-250-s001.docx]

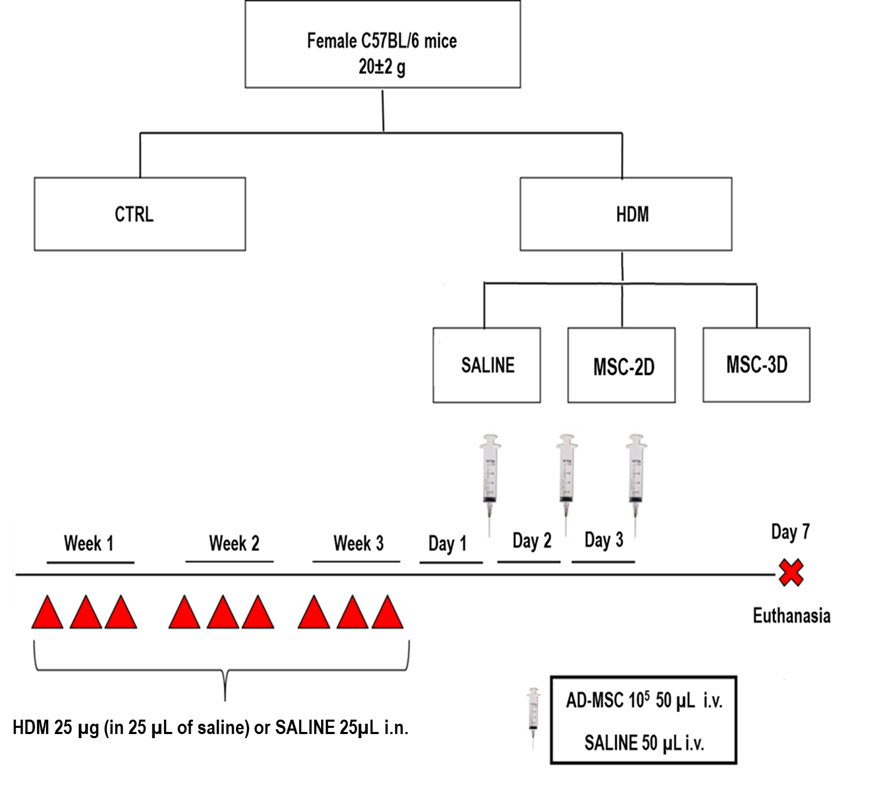


**Supporting Information Figure S1**. Experimental design. Female C57BL/6 mice were randomly divided into 2 groups. CTRL group was challenged with 25 µL of saline intranasally (i.n.) 3 times a week for 3 weeks and HDM group received 25 µg of HDM diluted in saline (25 µL). The HDM group was then treated with saline (50 µL) for 3 consecutive days after the last challenge, or 2 or 3 doses of 10^5^ adipose tissue (AD) derived-MSC diluted in saline (50 µL) for 2 or 3 consecutive days after the last challenge (MSC-2D and MSC-3D, respectively). All treatments were administered intravenously. Seven days after the last challenge, the animals were euthanized for data acquisition.
