# Supplementary material for: Multiple doses of adipose tissue‐derived mesenchymal stromal cells induce immunosuppression in experimental asthma
Source: Stem Cells Transl Med. 2019 Nov 20;9(2):250–60. doi: 10.1002/sctm.19-0120 (PMC6988761; doi:10.1002/sctm.19-0120)
Supplement: Supplementary file 2 — Supporting Information Figure S2 Representative histogram with the gating strategy used to quantify CD4+ T‐cells (top) and eosinophils (bottom) in bronchoalveolar lavage fluid. [file SCT3-9-250-s002.docx]

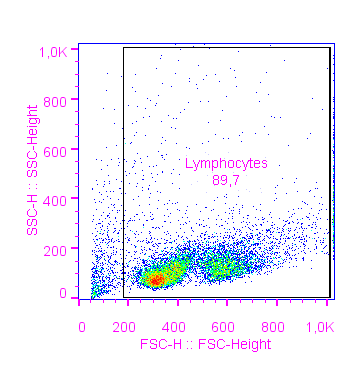

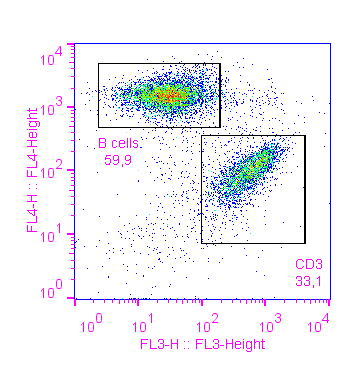

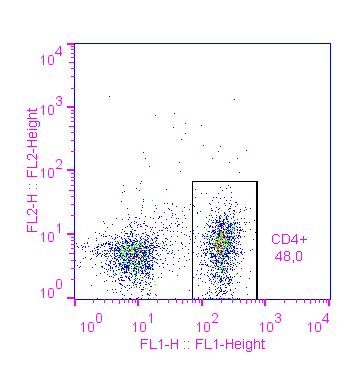

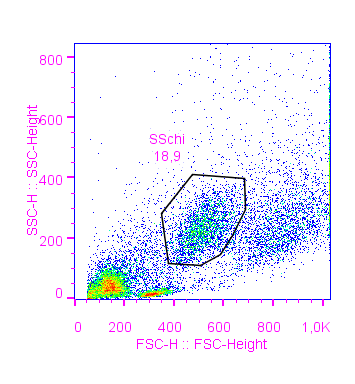

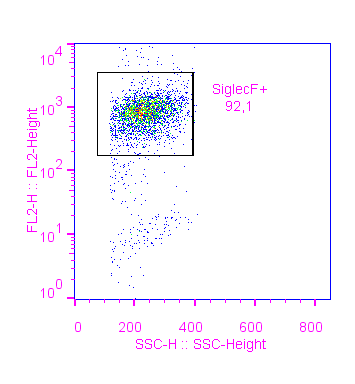


**Supporting Information Figure S2:** Representative histogram with the gating strategy used to quantify CD4+ T-cells (top) and eosinophils (bottom) in bronchoalveolar lavage fluid.
