# Supplementary material for: Multiple doses of adipose tissue‐derived mesenchymal stromal cells induce immunosuppression in experimental asthma
Source: Stem Cells Transl Med. 2019 Nov 20;9(2):250–60. doi: 10.1002/sctm.19-0120 (PMC6988761; doi:10.1002/sctm.19-0120)
Supplement: Supplementary file 3 — Supporting Information Figure S3 Relative weight of mediastinal lymph nodes and thymus. Data are presented as means + SD of 6 animals/group. SAL: mice challenged with HDM and treated with saline. MSC‐2D and MSC‐3D: mice challenged with HDM and treated with 2 or 3 doses of AD‐MSCs, respectively. * Significantly different from CTRL (P < 0.05). # Significantly different from SAL (P < 0.05). [file SCT3-9-250-s003.docx]

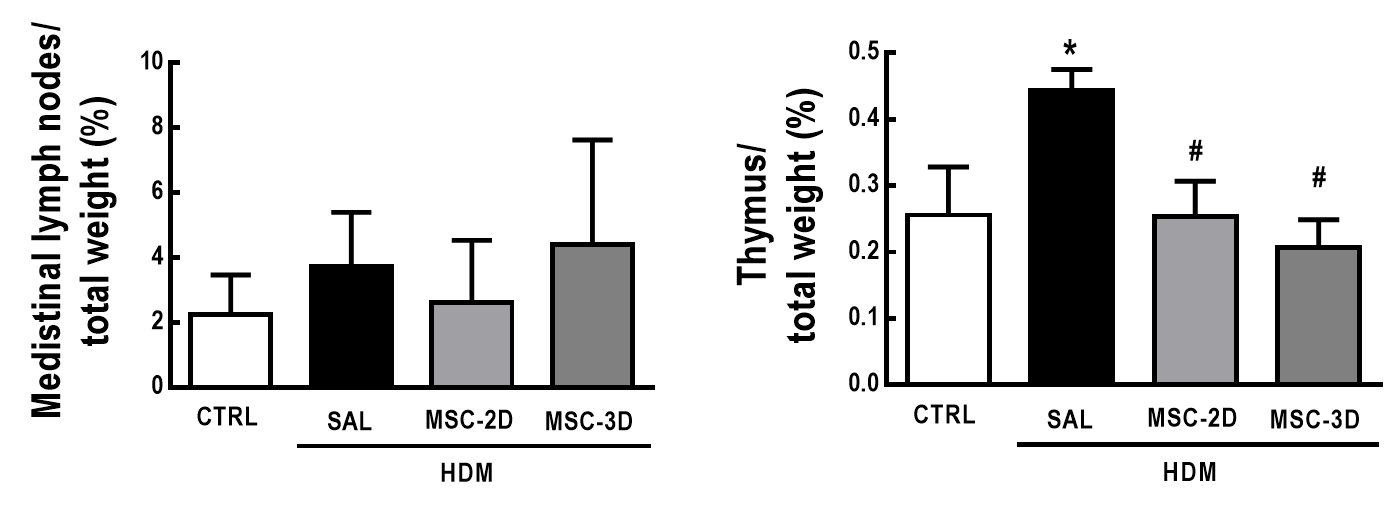


**Supporting Information Figure S3:** Relative weight of mediastinal lymph nodes and thymus. Data are presented as means + SD of 6 animals/group. SAL: mice challenged with HDM and treated with saline. MSC-2D and MSC-3D: mice challenged with HDM and treated with 2 or 3 doses of AD-MSCs, respectively. * Significantly different from CTRL (p<0.05). # Significantly different from SAL (p<0.05).
