# Supplementary material for: Multiple doses of adipose tissue‐derived mesenchymal stromal cells induce immunosuppression in experimental asthma
Source: Stem Cells Transl Med. 2019 Nov 20;9(2):250–60. doi: 10.1002/sctm.19-0120 (PMC6988761; doi:10.1002/sctm.19-0120)
Supplement: Supplementary file 4 — Supporting Information Figure S4 Dexamethasone therapy and three doses of MSCs modulated protein levels of programmed death receptor (PD)‐1, and interleukin (IL)‐10 in the thymic tissue of animals with HDM‐induced allergic asthma. CTRL, mice challenged with saline and treated with saline. HDM‐SAL, mice challenged with HDM and treated with saline. HDM‐DEXA, mice challenged with HDM and treated with dexamethasone. HDM‐MSC‐2D, mice challenged with HDM and treated with two doses of MSCs (105 cells per dose). HDM‐MSC‐3D, mice challenged with HDM and treated with three doses of MSCs. Data are presented as means ± SD of 6 animals/group. *Significantly different from CTRL (P < 0.05). #Significantly different from HDM‐SAL (P < 0.05). †Significantly different from HDM‐DEXA (P < 0.05). ‡Significantly different from HDM‐MSC‐2D (P < 0.05). [file SCT3-9-250-s004.docx]

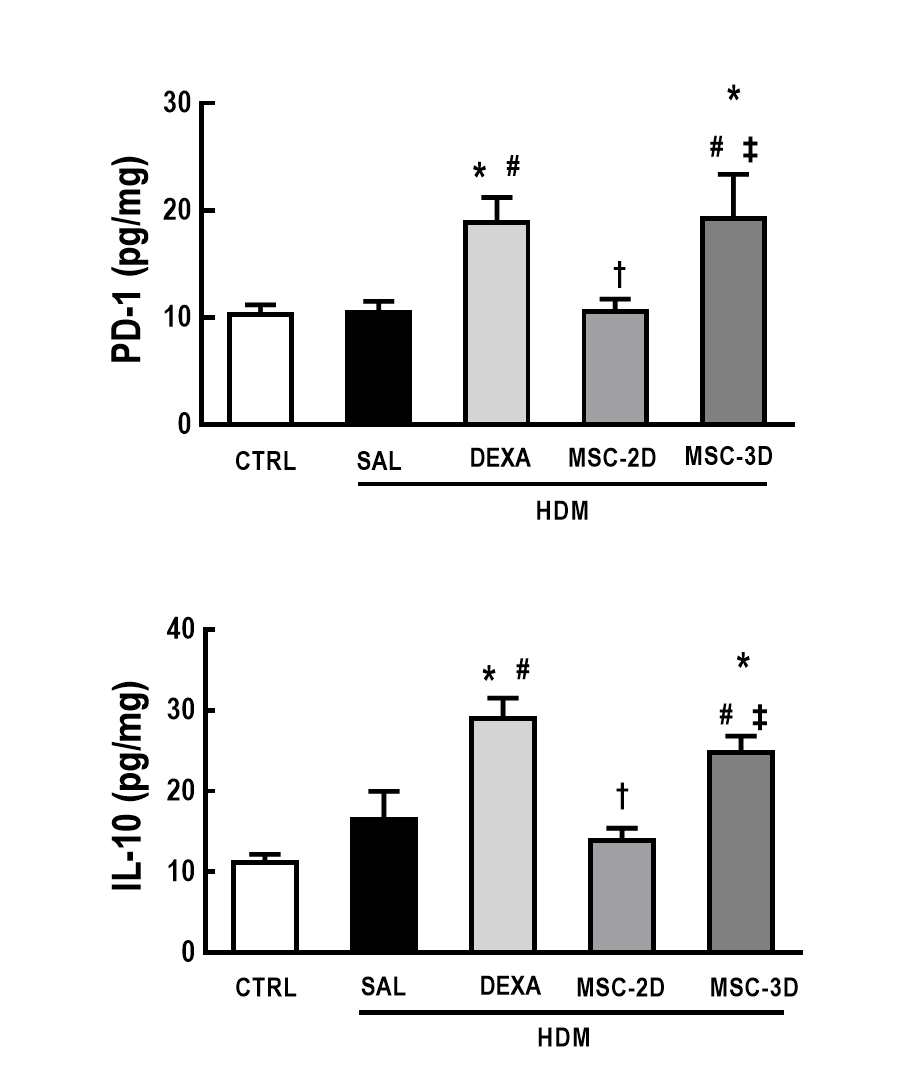


**Supporting Information Figure S4:** Dexamethasone therapy and three doses of MSCs modulated protein levels of programmed death receptor (PD)-1, and interleukin (IL)-10 in the thymic tissue of animals with HDM-induced allergic asthma. CTRL, mice challenged with saline and treated with saline. HDM-SAL, mice challenged with HDM and treated with saline. HDM-DEXA, mice challenged with HDM and treated with dexamethasone. HDM-MSC-2D, mice challenged with HDM and treated with two doses of MSCs (10^5^ cells per dose). HDM-MSC-3D, mice challenged with HDM and treated with three doses of MSCs. Data are presented as means ± SD of 6 animals/group. *Significantly different from CTRL (P<0.05). ^#^Significantly different from HDM-SAL (P<0.05). ^†^Significantly different from HDM-DEXA (P<0.05). ^‡^Significantly different from HDM-MSC-2D (P<0.05).
