# Supplementary material for: Multiple doses of adipose tissue‐derived mesenchymal stromal cells induce immunosuppression in experimental asthma
Source: Stem Cells Transl Med. 2019 Nov 20;9(2):250–60. doi: 10.1002/sctm.19-0120 (PMC6988761; doi:10.1002/sctm.19-0120)
Supplement: Supplementary file 5 — Table S1 Sequence of PCR primers [file SCT3-9-250-s005.docx]

**Supporting Information**

**Table S1 –** Sequence of PCR primers

| Gene | Forward | Reverse |
| --- | --- | --- |
| IDO-2 | 5′**–**AGA CCA CCA CAT AGA TGA AG**–**3′ | 5′**–**CCA CCA ATA GAG AGA CGA GGA**–**3′ |
| CD39 | 5′**–**AAG GTG AAG AGA TTT TGC TCC AA**–**3′ | 5′**–**TTT GTT CTG GGT CAG TCC CAC**–**3′ |
| Galectin | 5′**–**AAC CTG GGG AAT GTC TCA AAG T**–**3′ | 5′**–**GGT GAT GCA CAC CTC TGT GA**–**3′ |
| CTLA-4 | 5′**–**GGG TGT TTC ATG TGC TGT TG**–**3′ | 5′**–**AGT TTC CAA GCC AGT TGG TG**–**3′ |
| PD-1 | 5′**–**CGT CCC TCA GTC AAG AGG AG**–**3′ | 5′**–**GTC CCT AGA AGT GCC CAA CA**–**3′ |
| IL-10 | 5′**–**ATC CAA GAC AAC ACT ACT ATA**–**3′ | 5′**–**TAA ATA TCC TCA AAG TTC C**–**3′ |
| 36B4 | 5′**–**CAA CCC AGC TCT GGA GAA AC**–**3′ | 5′**–**GTT CTG AGC TGG CAC AGT GA**–**3′ |

IDO, indoleamine 2,3-dioxygenase; CTLA, cytotoxic T-lymphocyte-associated antigen; PD, programmed death receptor; IL, interleukin; 36B4, acidic ribosomal phosphoprotein P0.
