# Supplementary material for: Multiple doses of adipose tissue‐derived mesenchymal stromal cells induce immunosuppression in experimental asthma
Source: Stem Cells Transl Med. 2019 Nov 20;9(2):250–60. doi: 10.1002/sctm.19-0120 (PMC6988761; doi:10.1002/sctm.19-0120)
Supplement: Supplementary file 6 — Table S2 Body weight of animals (g) before and after HDM‐induced allergic asthma and therapeutic protocol [file SCT3-9-250-s006.docx]

**Supporting Information**

**Table S2 –** Body weight of animals (g) before and after HDM-induced allergic asthma and therapeutic protocol

| Groups | Before | After |
| --- | --- | --- |
| CTRL | 18.2±0.1 | 21.0±1.0 |
| HDM-SAL | 18.4±0.4 | 21.4±0.9 |
| HDM-MSC-2D | 18.0±0.3 | 21.4±0.5 |
| HDM-MSC-3D | 18.2±0.2 | 20.3±0.8 |

CTRL: mice challenged with saline solution and treated with saline for 3 consecutive days. HDM-SAL: mice challenged with HDM solution and treated with saline for 3 consecutive days. HDM-MSC-2D: mice challenged with HDM solution and treated with two doses of MSCs (10^5^ cells per dose). HDM-MSC-3D: mice challenged with HDM solution and treated with three doses of MSCs (10^5^ cells per dose).
