# Supplementary material for: Multiple doses of adipose tissue‐derived mesenchymal stromal cells induce immunosuppression in experimental asthma
Source: Stem Cells Transl Med. 2019 Nov 20;9(2):250–60. doi: 10.1002/sctm.19-0120 (PMC6988761; doi:10.1002/sctm.19-0120)
Supplement: Supplementary file 7 — Table S3 T‐cell subpopulations in the thymus [file SCT3-9-250-s007.docx]

**Supporting Information**

**Table S3 –** T-cell subpopulations in the thymus

|  | CD4^-^ CD8^-^ | CD4^+^ CD8^+^ | CD4^+^ CD8^low^ | CD4^low^ CD8^+^ | CD4^+^ CD8^-^ | CD4^-^ CD8^+^ |
| --- | --- | --- | --- | --- | --- | --- |
| CTRL | 3.62±0.6 | 53.13±4.7 | 21.23±3.0 | 1.61±0.2 | 1.52±0.3 | 10.04±1.5 |
| HDM-SAL | 4.36±0.9 | 59.76±13.0 | 18.56±6.7 | 1.84±0.7 | 1.18±0.4 | 8.98±0.8 |
| HDM-DEXA | 5.09±0.8 | 69.94±11.3* | 6.16±2.0*^,#^ | 1.45±0.3 | 1.00±0.4 | 3.32±0.4*^,#^ |
| HDM-MSC-2D | 4.14±1.4 | 60.25±7.2 | 13.44±7.6 | 1.88±0.6 | 1.65±0.6 | 8.60±0.7^†^ |
| HDM-MSC-3D | 4.41±0.6 | 70.40±6.3* | 7.78±3.1*^,#^ | 2.00±0.3 | 1.74±0.5 | 9.53±1.6^†^ |

CTRL: mice challenged with saline solution and treated with saline for 3 consecutive days. HDM-SAL: mice challenged with HDM solution and treated with saline for 3 consecutive days. HDM-DEXA: mice challenged with HDM solution and treated with dexamethasone (1 mg/kg) for 3 consecutive days. HDM-MSC-2D: mice challenged with HDM solution and treated with two doses of MSCs (10^5^ cells per dose). HDM-MSC-3D: mice challenged with HDM solution and treated with three doses of MSCs (10^5^ cells per dose). *Significantly different from CTRL (p<0.05). ^#^Significantly different from HDM-SAL (p<0.05). ^†^Significantly different from HDM-DEXA (p<0.05).
